# Embracing the psychological complexity of tinnitus-correlated distress: from descriptive nosology to person-centred explanatory models

**DOI:** 10.3389/fpsyt.2025.1724030

**Published:** 2026-01-27

**Authors:** Benjamin Boecking, Kurt Steinmetzger, Matthias Rose, Petra Brueggemann, Birgit Mazurek

**Affiliations:** 1 Tinnitus Centre, Charité – Universitatsmedizin Berlin, Berlin, Germany; 2 Medical Department, Division of Psychosomatic Medicine, Charité – Universitatsmedizin Berlin, Berlin, Germany

**Keywords:** chronic tinnitus, clinical psychology and health, diagnosis criticism, HiTOP, psychological formulation, psychological therapy, person-centred psychological treatment

## Abstract

Chronic tinnitus presents a psychosomatic paradox: while the perceptual characteristics of the sound are often similar across individuals and typically linked to hearing loss, the distress it evokes differs substantially. This distress predicts the experiential quality, persistence, and chronification of tinnitus perception, thereby shaping what is recognised as chronic tinnitus or tinnitus disorder in clinical practice. In conceptualizing distress, two interconnected processes of medicalisation can be observed: first, the framing of distressing tinnitus as a medical disorder in its own right; and second, the framing of distress itself as a psychiatric “comorbidity.” However, the dominant medical-descriptive frameworks in psychiatry that underpin these practices rely on atheoretical, categorical systems to classify emotional distress. These systems have well-documented limits in reliability, validity, and clinical utility and tend to privilege biomedical explanations over psychosocial context. At this juncture, the current paper examines three conceptual approaches to distress: (1) medical-nomothetic-descriptive, (2) psychological-nomothetic-descriptive, and (3) psychological-idiographic-descriptive — which becomes explanatory upon considering individuals’ life contexts, meaning-based appraisals, and symptom functions. For both clinical and research practice in chronic tinnitus, we argue for a shift towards psychological-explanatory-idiographic models that account for person-specific interactions of vulnerability, stress , emotions, and coping (VSEC), linked through personal meaning. This aligns with a broader momentum towards idiographic, process-based therapies as the future of psychological intervention. Although meaning-centred formulations challenge nomothetic research methodologies, they offer clearer clinical reasoning and help avoid unhelpful medicalisation beyond somatic factors which contribute to initial symptom onset.

## Introduction

Chronic tinnitus, i.e. “*the conscious awareness of a tonal or composite noise for which there is no identifiable corresponding external acoustic source*” (1, p. 1) affects millions worldwide. While its auditory characteristics (e.g., pitch or loudness) are often similar across patients, the degree of associated distress varies markedly, ranging from negligible to severely disabling. While onset is commonly linked to peripheral hearing loss (2), it is psychological factors that prospectively predict the persistence of tinnitus awareness and distress (3, 4). Epidemiological data further underscore this dissociation: whilst many people experience tinnitus at some point, only 7% of individuals report significant distress, with around 2% requiring clinical intervention (5).

To advance clinical understanding of ongoing tinnitus perception and enhance psychosocial treatment approaches, it is thus essential to adopt conceptual frameworks that account for the nature and maintenance of emotional distress. This shift involves moving the central clinical question away from “Why is *tinnitus* distressing?” towards “*Why* is tinnitus distressing *for this person*?”. To address this question, models of distress can be organised along three intersecting axes:

1. **Medical - psychological models** — contrasting views of distress as manifestations of psychiatric illness (rooted in biological malfunction) with views of distress as a meaningful phenomenon shaped by personal and contextual variables (6);
2. **Nomothetic – idiographic models** — contrasting population-level patterns (variable-centred, between-person, quantitative research approaches) with individual, single-person configurations (person-centred, qualitative research approaches; 7, 8);
3. **Descriptive - explanatory models** — contrasting frameworks that *describe* features of distress as compared to frameworks that seek to understand *why* it arises. To orient readers, we briefly exemplify each axis (see **Table 1** for an overview).

**Table 1:** Eight perspectives on distress.

Level	Lens	Descriptive (What is)?	Explanatory (Why is)?
Nomothetic (general patterns)	Biomedical	“Major Depressive Disorder (MDD) with comorbid tinnitus”	“Peripheral hearing loss with central gain is associated with tinnitus perception; inflammatory and stress-system markers are elevated in groups with MDD.”
	Formulation-based psychological	“Individuals with low mood frequently report high levels of tinnitus-related distress.”	“Catastrophic appraisals, attentional hypervigilance, and experiential avoidance maintain both tinnitus distress and depressive symptom load.”
Idiographic (unique patterns)	Biomedical	“This person presents with high-frequency sensorineural hearing loss and symptoms consistent with a diagnosis of a depressive episode (low mood, insomnia, concentration difficulties). Further assessment is required to determine whether diagnostic criteria for MDD, single episode, mild are met (≥5 symptoms for ≥2 weeks, clinically significant distress/impairment, exclusions).”	“This person’s audiological profile suggests cochlear damage contributing to initial tinnitus symptom onset; her sleep fragmentation may be exacerbated by thyroid dysfunction.”
	Formulation-based psychological	“This person describes pronounced sadness, fatigue even after small efforts, rumination (mostly about memories of her father), and heightened tinnitus salience since her father’s death.”	“Against a history of ambivalent attachment and unresolved grief, she appraises the tinnitus as an accusing reminder of her father (“as if he is still screaming at me”), which evokes fear, anger, and guilt. These states amplify both her monitoring of the tinnitus to protect herself and her overall distress.”

The present paper argues that categorical nosologies have substantial shortcomings and that dimensional nosologies, while descriptively useful, remain largely silent on *why* a given person is distressed (e.g., by tinnitus). We therefore propose a shift in research and clinical practice towards person-centred, meaning-based explanatory models—exemplarily operationalised here via a Vulnerability–Stress–Emotion–Coping (VSEC) framework that integrates subjective meaning to guide psychological assessment and intervention. In this context, meaning denotes *the person-specific significance that an event, sensation, relationship, or symptom holds for a particular individual, given their developmental history, goals/values, identity, and current relational context. It is expressed in appraisals (“what this implies for me”), and in the functions which emotions and behaviors serve (e.g., to protect attachment, preserve control, signal injustice, avoid shame). It can be explicit (verbalizable beliefs) or implicit (felt tendencies, action patterns), and it is context-dependent and revisable*.

The paper offers an illustrative review of influential models in clinical psychology and psychiatry, selected for either their prominence in current medical discourse or their theoretical/clinical relevance to the nature and maintenance of tinnitus-related distress. We focus on three conceptual approaches: (1) medical–nomothetic–descriptive, (2) psychological–nomothetic–descriptive, and (3) psychological–idiographic–descriptive. Crucially, approach (3) becomes psychological–idiographic–explanatory once a person’s context, meaning-based appraisals, and the functions of symptoms are explicitly considered. For completeness, we briefly note the existence of a psychological–nomothetic–explanatory framework, although its details, like those of medical–explanatory perspectives, lie beyond the scope of this paper^1^.

## Tinnitus-correlated distress

Because conceptual frameworks guide both inference and action, the prevailing model in clinical practice—whether general or tinnitus-specific—functions as an organising principle for care, shaping how tinnitus-related distress is understood, communicated, and treated. Currently, tinnitus-correlated distress is often viewed through a medical–nomothetic–descriptive lens. However, while hearing loss is a frequent antecedent (commonly managed with hearing aids), the tinnitus percept lacks a medical etiology that accounts for the marked variability in distress across individuals (9). Nevertheless, the emotional burden associated with tinnitus is commonly medicalised as psychiatric comorbidity, anchored in standardised diagnostic criteria that describe symptom constellations, duration thresholds, and functional impairment. These conceptualisations—drawn from the **International Classification of Diseases** (Chapter F, ICD; 10) or the **Diagnostic and Statistical Manual of Mental Disorders** (DSM; 11)—appear to offer a shared descriptive vocabulary, yet label concordance frequently masks heterogeneity of mechanisms and person-specific meanings between individuals—a limitation reflected in critiques of reliability, validity, and clinical utility (12, 13). Most importantly, the diagnostic systems are largely *atheoretical*—describing symptoms without explaining the contexts in which they emerge or persist—thus offering limited insight into *why* tinnitus is distressing for some individuals but not others, and providing little guidance for individualised treatment approaches.

Consequently, research or clinical communication that frames ongoing, distressing tinnitus perception as “neurological illness” with “psychiatric comorbidity” risks obscuring the mechanisms that constitute and maintain emotional suffering.

A more empirically grounded alternative comes from psychological–nomothetic–descriptive models, which move away from categorical diagnoses towards dimensional understandings of psychopathology. Transdiagnostic frameworks (e.g., the Hierarchical Taxonomy of Psychopathology, HiTOP) aim to identify and validate psychological dimensions—expressed in signs, symptoms, and traits—that cut across traditional diagnostic boundaries. Existing evidence suggests that dimensional approaches can offer a more nuanced and clinically applicable description of symptom clusters and distress. However, like categorical nosologies, dimensional models remain descriptive and atheoretical: they reveal which traits or symptoms co-occur at the population level, not *why* they emerge or become distressing for a given person.

To address this central question—why tinnitus becomes distressing for some individuals but not others—the present paper turns to psychological–idiographic–explanatory frameworks, which aim to understand how individuals interpret, experience, and respond to tinnitus in light of their personal histories, vulnerabilities, relational contexts, and sociocultural environments—that is, the meaning they attach to the symptom. Though narrative in form, such "case formulations"—person-specific hypotheses that link history, context, appraisals, emotions, and behaviour—are grounded in psychological theory and clinical reasoning. They integrate cognitive, emotional, and interpersonal processes with meaning-making. When informed by subjective appraisals, idiographic models move beyond symptom description and offer explanatory insight, facilitate individualised treatment planning, and help avoid unnecessary medicalisation of psychosocial distress.

The current paper thus

1. introduces and critiques the dominant medical-nomothetic-descriptive framework in tinnitus research and clinical care,
2. considers HiTOP as a psychological-nomothetic-descriptive alternative,
3. outlines the limitations of applying group-level findings to individuals,
4. introduces the VSEC model as a flexible, though not comprehensive, person-centred psychological–idiographic-descriptive framework, which can become explanatory through the integration of personal meaning, and
5. discusses implications for clinical practice and research.

## Why is a person distressed?

### The medical-nomothetic-descriptive perspective

Chronic tinnitus is frequently framed as co-occurring with psychiatric comorbidity—most commonly “major depressive disorder” or “anxiety disorders” (14, 15). Such classificatory labels derive from categorical diagnostic taxonomies, including the ICD and DSM. These taxonomies reflect a medical–nomothetic–descriptive approach to psychopathology—which sorts distress into discrete categories via standardised clusters of symptoms, duration thresholds, and impairment criteria. By design, they *describe* syndromes rather than explain *why* a person is distressed

#### Limitations of psychiatric nosologies

Psychiatric diagnoses are often treated as if they referred to discrete disease states with biological underpinnings—even in the absence of identified pathophysiology (16). Beyond scientific and clinical concerns, this reflects a deeper ontological assumption embedded in psychiatric classification: that emotional distress is best understood as a “disorder” that a person somehow “has”. This transformation of experience into a quasi-medical “thing” risks detaching distress from the person’s lived experience, biographical background and relationships. The medicalised framing further encourages a model of the self where cognitions and emotions are thought of as alien, symptom-like phenomena intruding upon an otherwise stable subject, rather than meaningful responses to one’s life circumstances or adversity (17–19). Clinically, this may result in treatment approaches that prioritize symptom management over understanding personal meaning and interrelations of contexts, thoughts, emotions and behaviours (20, 21).

Despite their widespread use and considerable institutional influence, psychiatric diagnoses have long been subject to substantial critique regarding their *reliability*, *validity*, and *clinical utility*.

*Reliability*—the consistency of diagnostic outcomes across clinicians and settings—remains limited, and inter-rater agreement is often modest, particularly under ecologically valid conditions (22–24). For example, a study by Chmielewski et al. (25) found that the average inter-rater reliability for DSM diagnoses that were given in near-real-world settings (two raters, separate interviews) was only *κ ≈ 0.47* (“poor” to “fair”), whereas previous studies may have inflated reliability estimates at the cost of external validity by using two raters assessing one interview. Notably, test–retest reliability has been found to be particularly low for diagnoses frequently observed in tinnitus populations, such as major depressive disorder and generalised anxiety disorder (26).

*Validity*—the degree to which diagnostic categories correspond to actual, naturally occurring phenomena—is similarly problematic. High comorbidity rates, poorly demarcated diagnostic boundaries, and arbitrary diagnostic thresholds challenge the assumption that psychiatric categories reflect real disease entities (12, 27–30). Critics argue that the artificiality and constructed nature of these categories are often overlooked in clinical practice, with far-reaching consequences following from the assumption that psychiatric diagnoses represent natural disease states (31). Faravelli et al. (32, p. 12) note: “*the conditions that are currently classified as psychiatric disorders still appear as vague and confusing (rather than discrete) entities”* whilst Nemeroff et al. (33) concede that *“the [diagnostic] approach was never meant to establish disease validity”.*

A further challenge is the heterogeneity within diagnoses: many categories allow for a wide range of symptom combinations that lead to the same label. For example, two individuals diagnosed with major depressive disorder may share few, if any, symptoms—apart from the bare minimum needed for giving the diagnosis (34) — prior to even considering differences in psychological or contextual circumstances. This heterogeneity dilutes construct coherence, complicates validation efforts, and limits the generalisability of findings across individuals. Clinically, it also raises an important dilemma: if individuals sharing the same diagnosis differ substantially in symptom presentation, psychosocial context, and underlying meaning structure, it becomes impossible to generalise treatment from diagnosis. This may help explain the limited effectiveness observed in both psychopharmacological interventions (35–37) and disorder-specific elements of psychological treatments (38).

These conceptual and structural issues are compounded by the lack of objective biological markers or identified etiologies for most psychiatric disorders (39). As Hyman (40) cautions, diagnostic labels often come to be treated as “real” diseases by mere force of habit, even when their scientific underpinnings remain weak—potentially impeding the development of more valid, better informed constructs.

Finally, psychiatric diagnoses are not typically derived from empirical clustering of symptoms, but through expert consensus (41)—a process which is subject to considerable political, financial and institutional influences (42, 43).

*Clinical utility*—Concerns about the validity of diagnostic categories are further reflected in their limited utility in clinical practice. Whilst proponents argue that diagnoses offer benefits such as a “common language for describing a case” or “foundations for treatment decisions akin to medical diagnoses” (12), it is arguable whether such decisions might not be better guided by individualised formulations of patients’ presenting difficulties. Rather than focusing on symptom clusters alone, formulation seeks to understand the psychological function and meaning of a person’s difficulties within their intra-individual, interpersonal, biographical, and biopsychosocial context (20, 34).

These limitations in clinical utility are particularly evident in psychotherapeutic contexts where a strong therapeutic alliance often hinges on understanding a person’s unique narrative and symptoms’ *function*—rather than symptoms “*per se”*. To this end, clinicians point out that a rigid application of diagnosis labels and disorder-focused therapeutic approaches encourage a “checklist” style of interaction and intervention—which risks distancing clinician and patient and fails to grasp key clinical principles. Importantly, relying on diagnosis neither provides an explanation for a patient’s symptoms nor information about their individual experience and may thus poorly guide intervention choices (34).

Even if applied reliably, diagnoses rarely offer meaningful guidance for treatment planning, fail to predict prognosis or care duration, and often diverge from how individuals themselves understand or articulate their difficulties (20, 44–46). Moreover, meta-analytic evidence suggests that therapeutic outcomes are more strongly influenced by common factors—such as empathy, attunement, alliance and therapist characteristics—than by diagnosis-specific techniques (47–49)—leading to the reassertion that psychotherapy is foremost a relational endeavour, not a quasi-medical treatment enterprise.

#### The social perception of emotional distress

Beyond limited clinical guidance, psychiatric diagnoses can also shape how emotional distress is socially perceived and relationally used. For example, diagnoses may contribute to stigmatisation, relational distancing, or patronisation by framing distressed individuals as “ill” or “disordered” (19, 50, 51). By contrast, some individuals actively seek psychiatric diagnosis—possibly attempting to secure what they experience as an explanation (rather than a mere description) of their emotional distress (52–54) — or to search for recognition, support, category membership, sympathy, and a sense of belonging in a medicalised social landscape (55–59).

#### Mistaking description for explanation

Even if psychiatric diagnoses were reliable and valid, they would remain descriptive tools, not explanatory constructs. As Maung (16, p. 517) notes: *“If psychiatric diagnoses are mere labels for clusters of symptoms, then they cannot refer to the causes of these symptoms … [Nonetheless] psychiatric diagnoses are sometimes communicated to clinicians, patients, and the public as if they are causal explanations of patients’ symptoms, much like the diagnoses in [ … ] medical* sp*ecialties”.*

This misinterpretation is pervasive. Statements such as “X is distressed by tinnitus because of depression” invert the intended diagnostic logic, treating the label as the cause rather than a description of the symptoms it summarises. This fallacy not only fosters conceptual confusion, but also diverts clinical attention from understanding distress as a response to context, meaning, or adversity (17).

### Towards a dimensional alternative

In light of these scientific, clinical, and philosophical limitations, psychological science has begun to move from categorical approaches towards dimensional models of psychopathology. These models aim to empirically identify and organise naturally co-occurring signs, traits, and symptoms without presuming discrete disorders. The following section introduces one such approach—the **Hierarchical Taxonomy of Psychopathology** (HiTOP)—as a nomothetic, empirically grounded, and psychologically informed alternative to psychiatric taxonomies. Whilst still descriptive, HiTOP offers a more flexible, data-driven framework for characterizing emotional distress—and might become helpful in complex, heterogeneous contexts which surround chronic tinnitus as index symptom (60).

### A psychological-nomothetic-descriptive perspective

**Figure 1:**
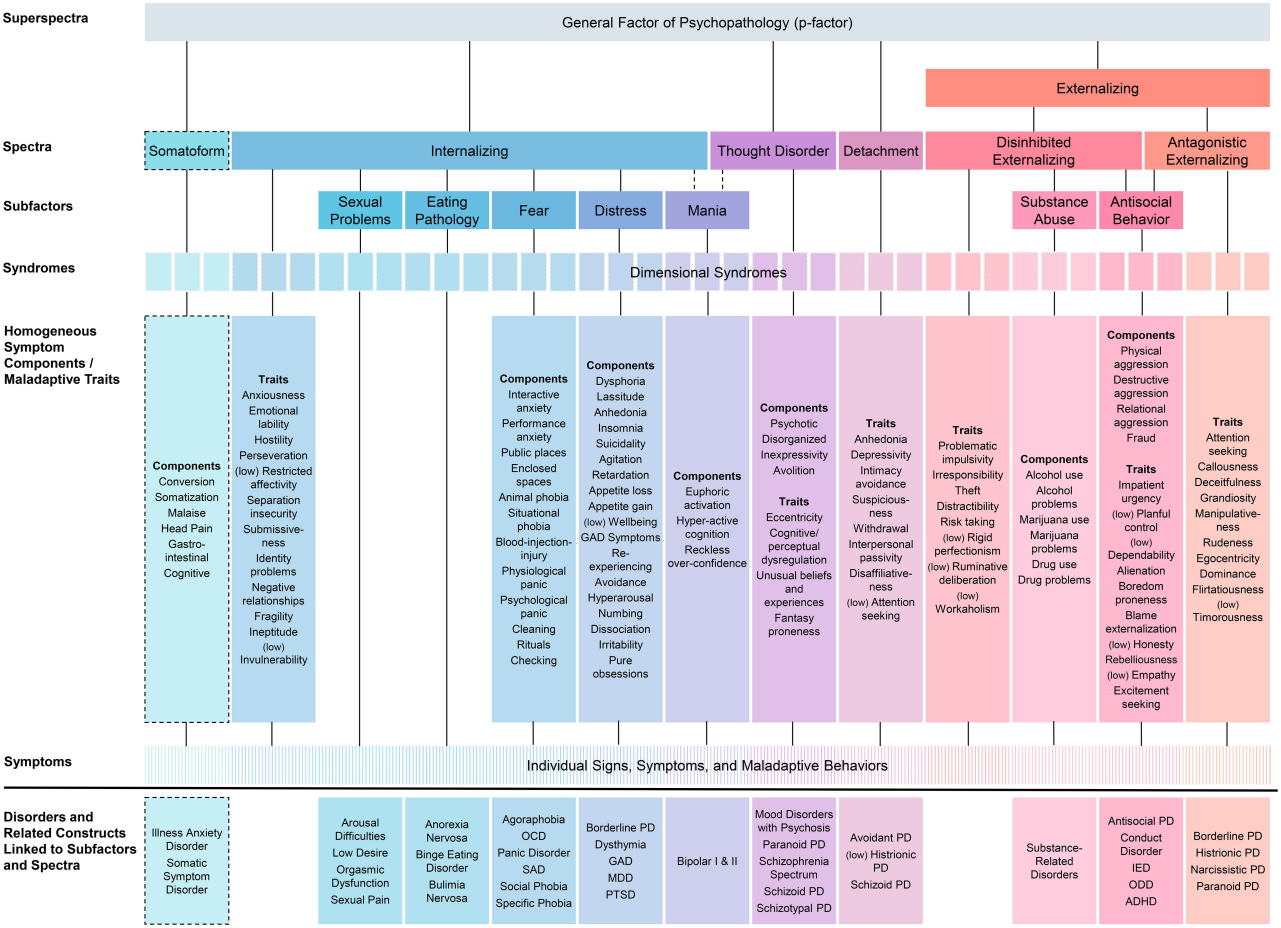
The Hierarchical Taxonomy of Psychopathology (HiTOP) Reproduced from (62) with permission of Cambridge University Press. Dashed lines indicate dimensions included on a provisional basis, as data on them are limited. Qualifier ‘(low)’ in front of a construct indicates negative relationship with the corresponding spectrum. DSM diagnoses are not included in HiTOP; rather symptoms and signs that constitute them are; also, diagnoses have been used in research to identify HiTOP subfactors and spectra. HiTOP syndromes are empirically derived dimensions. ADHD, attention-deficit/hyperactivity disorder; DSM, Diagnostic and Statistical Manual of Mental Disorders; GAD, generalised anxiety disorder; IED, intermittent explosive disorder; MDD, major depressive disorder; OCD, obsessive–compulsive disorder; ODD, oppositional defiant disorder; SAD, separation anxiety disorder; PD, personality disorder; PTSD, posttraumatic stress disorder.

To address limitations of categorical nosologies, a consortium of psychologists and psychiatrists developed a quantitative, data-driven system for describing psychopathology: HiTOP (61; see **Figure 1**). The framework, which is being continuously refined, aims to empirically identify dimensions of naturally co-occurring symptoms, maladaptive traits, and behaviours, grouping them hierarchically into broader spectra of psychopathology. These spectra thus reflect empirically identified patterns of symptom co-occurrence across a variety of populations (63) and the dimensional nature of psychological traits (64).

**Figure 1** illustrates HiTOP’s “bottom-up” structure. At the base of the hierarchy lie individual symptoms—specific signs, behaviours, or experiences indicative of psychological distress, such as a racing heartbeat when speaking to others, or frequent mood swings. These symptoms cluster into broader symptom components or maladaptive traits, such as performance anxiety or emotional lability. These, in turn, aggregate into syndromes—recurrently co-occurring combinations of symptom components. Syndromes are grouped into broader subfactors (e.g., fear, distress, disinhibition), which are themselves nested within overarching spectra or domains, such as Internalizing, Externalizing, or Thought Disorder. At the top of the hierarchy lies a general psychopathology factor (often referred to as the p-factor), which reflects an individual’s overall propensity towards psychological dysfunction (65).

So far, empirical research on the clinical utility and real-world uptake of HiTOP remains in its early stages—a limitation that is to be expected given the model’s relatively recent development and ongoing refinement (66, 67). However, initial data suggest that it may offer advantages over traditional categorical systems in key clinical domains. For example, Balling et al. (68) reported that clinicians judged the HiTOP model superior to the DSM in terms of its utility for case conceptualisation, client communication, psychopathology coverage, functional assessment, and overall usability. Moreover, HiTOP spectra were shown to outperform diagnoses in key outcome domains such as psychiatric and physical health outcomes, community functioning, neural responses, or mortality (69, 70).

### “Tinnitus disorder” and the HiTOP

To date, “tinnitus disorder”—defined as “*the conscious awareness of a tonal or composite noise for which there is no identifiable corresponding external acoustic source [and which is] associated with emotional distress, cognitive dysfunction, and/or autonomic arousal, leading to behavioral changes and functional disability*” (1)—has not been formally examined within the HiTOP framework. However, when existing evidence on the psychological characteristics of individuals with chronic tinnitus is considered in light of HiTOP, preliminary findings suggest potential relevance of the Somatoform, Internalizing, Thought Disorder, Detachment, and Disinhibited Externalizing spectra, as well as subfactors such as Fear, Distress, and Substance Misuse (71–74). Establishing such empirical links would represent a promising avenue for future research, potentially enriching both diagnostic clarity and intervention development.

### A psychological-nomothetic-explanatory perspective

While HiTOP offers a psychological-nomothetic-descriptive account, it does not (aim to) explain *why* psychological distress arises. One prominent nomothetic framework that aims to address this question is the so-called Power Threat Meaning Framework (PTMF; 18, 75)—which meta-conceptualises emotional distress as a consequence of experiences of powerlessness and threat which are interpreted through prisms of individual, cultural, and social meaning-making processes. Whilst conceptually rich and critically oriented, a recent scoping review highlighted the current lack of empirical evidence regarding its clinical utility (76). To date, “tinnitus disorder” has not been formally examined within the PTMF either. Nevertheless, the PTMF offers a promising framework for advancing understanding of the psychosocial dimensions of tinnitus-correlated distress and for guiding the development of related interventions. Accordingly, the PTMF should be revisited as future studies evaluate its clinical uptake and utility.

### The nomothetic–idiographic divide

Whilst dimensional approaches represent a significant advance over categorical diagnostic systems, they remain descriptive and nomothetic. As such, they are limited in their capacity to account for both inter- and intra-individual variability—dimensions that are critical for both clinical decision-making and psychological change (77). In practice, population-level findings that reflect what is effective “on average” often fail to translate meaningfully to the lived experience of a particular individual.

In contexts marked by high variability (60), statistically robust group effects often have limited clinical utility. This is evident in tinnitus-related distress: generic recommendations (e.g., “target attentional bias”) rarely explain or guide care for *this* patient, whose individual appraisals, coping strategies, and symptom functions may substantially differ from the population average.

Beyond this generalisability issue, descriptive models—whether categorical or dimensional—are further constrained by their inability to capture the functions, subjective meanings, and personal appraisals of symptoms. Across psychotherapeutic approaches, however, symptoms are not to be understood as isolated phenomena—but as meaningful reactions which are functionally embedded within past and current psychological contexts. This includes their roles in coping, identity, communication, and self-regulation, as well as the nuanced, sometimes contradictory ways in which individuals interpret and feel about their experience. Such complexity cannot be adequately represented by static, group-level descriptors alone.

A related concern is the persistent disconnect between medical models of mental health and relational models of psychotherapy (78). The medical model presupposes that accurate diagnosis followed by prescriptive treatment will resolve symptoms—an assumption much more applicable to physical illnesses with known pathophysiology than to psychological distress. This framing risks reducing clients to diagnoses and therapists to “treatment technicians”, overlooking the interpersonal, contextual, and dynamic nature of psychological functioning. Meta-analytic evidence consistently shows that outcomes are more strongly influenced by client factors, therapist characteristics, and the therapeutic relationship than by specific techniques linked to diagnoses (79–82). Consequently, effective care requires person-centred assessment and formulation—approaches that may or may not align with nomothetically derived protocols. This necessitates clinical flexibility, a broad repertoire of conceptual and technical tools, and an emphasis on therapeutic responsiveness rather than strict protocol adherence.

To navigate these complexities, it is essential to distinguish—and integrate—nomothetic generalisations and idiographic formulations. Population-level models can inform hypotheses, but individualised psychological care must be grounded in a person’s subjective appraisals, psychological history, and relational context. The following section introduces the **Vulnerability–Stress–Emotion–Coping (VSEC) model** as a framework for constructing idiographic, dynamic formulations of tinnitus-correlated distress—shifting from a descriptive to a clinically explanatory approach by placing subjective meaning at its core.

### A psychological-idiographic-descriptive perspective

Among psychologically informed conceptualisations of distress—with or without identifiable somatic correlates—we present the VSEC model as *one* flexible, idiographic framework for formulating psychological distress in chronic tinnitus (83, 84). Unlike fixed diagnostic systems, the VSEC model supports psychological formulation in that clinicians and patients co-construct a dynamic account of the person’s difficulties that remains open to meaning-based psychosocial interpretations (83). Importantly, the VSEC model is not “*the correct*” idiographic model; numerous formulation frameworks exist (e.g., cognitive–behavioural, narrative, systemic, psychodynamic, etc.). As with categorical diagnoses, the reliability of such formulations may also raise questions (85); however, in clinical practice it matters less whether different clinicians would arrive at an identical formulation and more whether the shared narrative makes sense to clinician and patient, helps them understand and anticipate experience and behaviour, and thereby supports the choice of psychologically valid interventions. The core elements of the VSEC model are:

1. Vulnerability: Developmental and relational experiences (and other predisposing factors) that shape a person’s sense of self, identity, and relational safety.
2. Stress: Current experiences—often interpersonal or intrapsychic—that threaten a person’s sense of self and activate underlying vulnerabilities. These may include, but are not limited to, the tinnitus symptom and are stressful for idiographic, initially unknown psychological reasons.
3. Emotion: Affective states that arise in response to "stress", together with their associated arousal (86). Clinically, many such states can be understood as organised around a small number of core emotional systems—such as fear/anxiety, anger, and sadness—even when described using a broader emotion vocabulary (e.g. grief, shame, guilt).
4. Coping: Internal or interpersonal attempts to manage and regulate emotions, often using ‘tried-and-tested’ strategies that previously served important psychological functions and may still be experienced as necessary or protective.

For example, nomothetically identified vulnerabilities for tinnitus-correlated distress such as traumatic life events (87), temperamental variables (88), or personality traits such as emotional excitability or aggression inhibition (89)—may predispose an individual to appraise the tinnitus sound as threatening or anger-provoking (90)—possibly by evoking negative autobiographical memories (91, 92). Resulting emotions may be managed via coping strategies such as increased alcohol use (93), aggressiveness, or emotional detachment (94)—which can inadvertently heighten monitoring of the tinnitus symptom and maintain emotional distress.

The VSEC model invites clinicians to consider (1) how nomothetic factors may (or may not) apply to a particular person and (2) which idiosyncratic contributions (biographical experiences, meanings, function of symptoms) shape their experience—especially aspects not captured by population-level research. In this way, VSEC supports a formulation-based approach which is informed, but not constrained, by group-level findings. Effective assessment thus necessitates respect for both the value and limits of nomothetic evidence, yet needs to include a disciplined focus on person-specific psychological nuance and emotional complexity.

### From descriptive to explanatory: the mediating role of meaning

Whilst the VSEC structure offers a helpful “sorting grid”, it risks being misapplied as a static “list-of-factors” framework—a descriptive summary that merely enumerates personally relevant variables without clarifying their psychological function or interrelation as viewed through the lens of *meaning*. Alas, clinical nuance and therapeutic leverage lie in understanding how certain influences *function* within a person’s life context. Crucially, it is the *psychological meaning* of experiences—not their objective presence or features alone—that mediates their emotional and behavioural impact (95, 96).

As Johnstone et al. (2018, p. 805) note: “*While research seeking very* sp*ecific causal pathways between adversity and outcomes has useful aspects, it fails to acknowledge that such pathways [ … ] in all likelihood cannot exist in relation to human thoughts, feelings, and behaviours. [ … T]he factors that contribute to any aspect of human behavior [ … ] are, crucially, always shaped by personal meaning and agency”*. For example, two individuals may report both tinnitus and early traumatic experiences, yet differ markedly in their psychological responses. In one case, the traumatic experience may have undermined the person’s sense of safety, agency, or identity—laying the groundwork for latent psychological themes such as helplessness, entrapment, or vulnerability. These underlying patterns may remain dormant until they are reactivated by the tinnitus symptom through associative processes, leading to intensified emotional distress and an increased likelihood of symptom chronicity. In another case, similar events may have been given a different meaning, integrated within more supportive relational contexts, or linked to different vulnerability themes; as a result, tinnitus does not activate the same latent patterns and may be experienced as a relatively neutral or merely annoying symptom.

Thus, meaning transforms a descriptive case model into a clinically explanatory narrative, which shapes both clinical understanding and intervention. From this perspective, tinnitus needs to be understood within an individual psychological system, and sustained symptom focus serves a function in managing inner challenges. For instance, a person may have learned early that vigilance and self-control keep them safe—often in contexts of high (emotional) unpredictability. They have to closely monitor moods and conflicts in order to maintain a sense of security. Later, this monitoring transfers to the tinnitus symptom which may be appraised as a “dangerous other” that must be watched to preserve senses of safety and control. Another person may experience tinnitus as a presence-in-silence, potentially helping them cope with feelings of loneliness. Still another person may experience it as an alarm indicating emotional tension linked to ambivalent or unprocessed feelings, which are themselves linked to potentially distressing memories or experiences. Clinically, it is crucial to understand what role tinnitus plays for one particular person’s sense of identity, relationships, sense of self-worth and emotion regulation. Once this meaning is understood, alternative ways of meeting the symptom’s function can be co-created—for example, safer ways to meet needs for connection or control.

**Figure 2:**
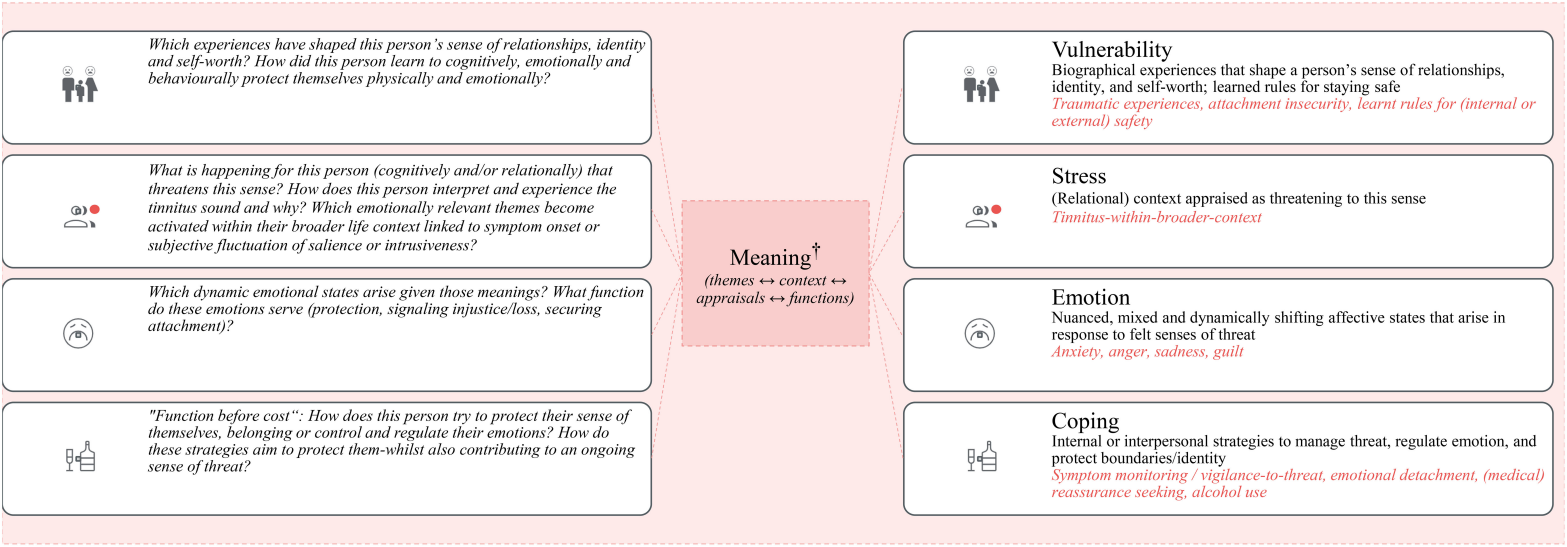
A Vulnerability–Stress–Emotion–Coping (VSEC) formulation. Tinnitus presents as *one* stressor within a wider, person-specific psychological system comprising developmental vulnerabilities, other current stressors, shifting emotional states and coping strategies. The **right-hand panel** shows an idiographic description of potentially relevant influences. However, it is only through meaning-oriented collaborative enquiry (psychological themes, appraisals, and the functions of emotions as well as “symptoms” and behaviours) that the map becomes explanatory; i.e. giving information about *why* distress arises and persists for this individual (**left-hand panel**). Importantly, the given prompts are not to be understood as “checklists”, but general domains for enquiry and hypothesis generation. Such hypotheses are developed tentatively, tested against lived experience, and revised together as the therapeutic relationship and process evolve. Questions are asked in a non-leading manner and with room for ambiguity and ambivalence; answers are expected to be layered, partial, and evolving. ^†^Meaning denotes the person-specific significance that an event, sensation, relationship, or symptom holds for a particular individual, given their developmental history, goals/values, identity, and current relational context. It is expressed in appraisals (“what this implies for me”), and in the functions that emotions and behaviours serve (e.g., to protect attachment, preserve control, signal injustice, avoid shame). It can be explicit (verbalisable beliefs) or implicit (felt tendencies, action patterns), and is context-dependent and revisable.

The clinical implications of this approach (“from checklist to narrative”) are profound: rather than diagnosing, cataloguing, or “treating” the symptom, clinicians ought to collaboratively construct psychologically informed, meaningful explanations that support flexible, individualised care. Recent psychological approaches, such as Process-Based Therapy, reflect the relevance of such a meaning-based stance and postulate that intervention should be centred on empirically tractable processes (not disorders) and their functional relations in a given individual (97, 98). **Figure 2** illustrates how the VSEC model can help to organise an individual’s difficulties and how the integration of subjective meaning turns this descriptive map into an explanatory formulation.

### Advancing psychological explanatory models: research pointers

One of the central challenges in developing meaning-informed psychological models lies in the operationalisation of subjective meaning in ways that support both empirical investigation and clinical generalisability. Psychological meaning is inherently dynamic, context-sensitive, and deeply personal—rendering it difficult to capture through conventional quantitative methodologies. In chronic tinnitus research, some qualitative studies have provided insight into the lived experience of distress (e.g. 91, 99, 100), whilst transdiagnostic, experience-focused dimensional measures have begun to examine putatively relevant psychological processes (71, 101)

However, existing studies are often limited in scale, and their broader applicability remains constrained by the elusive nature of psychological meaning and its embeddedness within fluid, interacting psychological systems. Expanding this research base is essential—but methodologically demanding. Whilst subjective meaning cannot be fully captured in quantitative terms, certain aspects of how people make sense of their experience may nonetheless be approximated through empirical markers. Recent developments in computational approaches offer promising pathways forward. For instance, machine learning techniques applied to mixed-method datasets may help identify latent meaning structures or narrative markers predictive of distress trajectories or treatment response. Such integrative approaches represent important progress towards capturing the complexity of meaning as it relates to psychological distress. Ultimately, advancing this line of work will be critical for developing more psychologically informed biopsychosocial interventions—moving beyond mechanistic or reductive operationalisations of distress towards empirically supported, individually meaningful treatment plans.

## Summary and conclusion

The present paper contrasts leading psychiatric and psychological frameworks for understanding emotional distress. We criticise prevailing medical-nomothetic-descriptive systems (DSM/ICD) and acknowledge that dimensional and empirically grounded psychological–nomothetic–descriptive alternatives (e.g., HiTOP) reflect crucial progress in the description of psychological distress. Both frameworks, however, remain silent about *why* a particular individual becomes distressed. Psychological models have the potential to offer finer descriptions of distress, but it is their explanatory, idiographic application—inquiring into meaning, appraisal, function, and context—that opens genuine understanding and, only then, guides psychological intervention. We operationalise this shift with the VSEC model, which needs to integrate personal meaning to connect vulnerabilities, stressors, emotional responses, and coping patterns at the person level. **Table 2** summarises each model and illustrates how a single case can be conceptualised under each framework.

**Table 2:** Exemplary case illustration: contrasting diagnostic, dimensional, and formulation-based idiographic approaches.

Ms. K (40) lives with chronic tinnitus whose intrusiveness varies across the day. Over the past three to four months she describes insomnia (sleep-onset and maintenance) and concentration difficulties, which she attributes mostly to the tinnitus symptom. During clinical evaluation, she furthermore reports persistent low mood, loss of interest/pleasure, and reduced appetite. Two contextual factors appear to contribute to the onset of her emotional distress: clearing the family flat after her mother moved to assisted living and a colleague’s sudden illness. Ms. K does not recall any previous episodes of depression or other psychiatric illnesses.				
Approach	Features	Problem description/profile	Intervention anchor	Treatment plan (example)
Medical-nomothetic-descriptive DSM/ICD	**Type**: Categorical (thresholded criteria sets) **How it’s built**: Systematic evidence reviews → APA/WHO working groups draft operational criteria → multi-site field trials for reliability & clinical utility → revision → formal approval **Unit of description**: Diagnoses (thresholded clusters) **What it’s good at**: Widely established labels **Mechanism stance**: Atheoretical/biopsychosocial; mechanism-agnostic **Reliability/validity**: Limited **Clinical utility**: Limited for treatment planning; risks mistaking description for explanation **Risk if misapplied**: Reification of invalid labels; over-medicalisation of experience **Best use**: Administration; coarse communication	ICD-10: F32.1 — Depressive episode, moderate (≈ ICD-11: 6A70.1), comorbid with chronic tinnitus (e.g., H93.1). DSM-5-TR: Major Depressive Disorder, single episode, moderate, comorbid with tinnitus	Diagnosis + guideline; tinnitus as index symptom and “comorbid depression”	Hearing aids, if applicable, and CBT for depression and/or tinnitus and/or insomnia. Alternatively, for depression, antidepressant medication by preference/access. If insomnia is prominent: Mirtazapine 7.5–15 mg nocte; Trazodone 25–100 mg nocte; Doxepin 8–25 mg nocte (low dose); Eszopiclone 1–3 mg nocte (time-limited, e.g., up to ~6 months). Choice guided by patient priorities and side-effect profile (e.g., weight/appetite with mirtazapine; anticholinergic load with doxepin; next-day sedation) and local protocols. Psychological components: psychoeducation, behavioural activation, sleep schedule, stimulus control, attention redirection strategies
Psychological-nomothetic-descriptive HiTOP	**Type**: Dimensional (hierarchical spectra) **How it’s built**: Large multi-sample analyses of symptom co-variation (factor/structural models); iteratively refined **Unit of description**: Dimensions/spectra with lower-order components **What it’s good at**: Descriptive reliability/validity; phenotyping; finer-grained measurement; hypothesis seeding **Mechanism stance**: Atheoretical by design (structure ≠ cause) **Reliability/validity**: Improved via data-driven structure and cross-study replication **Clinical utility**: Work-in-progress; potentially useful for sampling/stratification and group-level inferences; limited direct guidance for individual treatment plans **Risk if misapplied**: “Averages ≠ individual”; trait reification **Best use**: Research sampling; measure-guided hypotheses	Elevated on the Internalizing spectrum, especially Distress subfactor with moderate burden. No symptom expressions on the Externalizing or Thought-Disorder spectra.	Spectra (dimensional symptom profiles)	Transdiagnostically evaluated interventions for Internalizing/Distress expressions^†^
Psychological-idiographic-explanatory^‡^ VSEC	**Type**: Dimensional (within-person formulation) **How it’s built**: Case formulation linking Vulnerability–Stress–Emotion–Coping via personal meaning/functions (contextualised appraisals); idiographic (not aimed at nomothetic verification) **Unit of description**: Narrative network of assumed and theoretically informed psychological mechanisms linking a particular person’s appraisals, feelings and behaviours in past and present **What it’s good at**: If considering subjective meaning and complexity, explains why a particular person is distressed; specifies intervention targets (appraisal/function/coping/context) **Mechanism stance**: Explicit psychological functions & meanings; testable within-person hypotheses **Reliability/validity**: Depends on formulation quality or skill and idiographic data (N-of-1/qualitative) **Clinical utility**: High when meaning is formulated; guides individualised intervention and alliance **Risk if misapplied**: Becomes mere description (V,S,E,C list without meaning/function) → weak guidance **Best use**: Formulation and intervention planning; explains why tinnitus (or another stimulus) is distressing for a given individual when meaning is formulated; supports ongoing case testing/refinement	Ms. K describes feeling increasingly drained and emotionally numb over the past months. Mornings begin heavy and slow; getting started takes effort. She notices little appetite and often lets meals or messages pass. Energy wanes early, even after small tasks, and she finds it hard to keep pace with ordinary routines. Sleep is disrupted—she lies awake turning over unfinished thoughts—and her mind feels sluggish, especially when she must focus under pressure. On such days, tinnitus becomes more intrusive, filling the silence she tries to rest in. Socially, she has begun to increasingly withdraw, saying that even familiar and cherished pleasures “don’t quite reach (her).” Two current strains seem to deepen this weariness: clearing the family flat after her mother moved to assisted living, which stirred feelings of loss and duty for her, and a colleague’s sudden illness, which leaves her having to carry out extra tasks which she resents and feels guilty about.	Person-centred formulation - co-constructed with Ms. K’s meaning-making (not as a checklist of factors): ■ V: Early lessons that love and safety depended on being “good” and “useful”; anger was internalised as risky if she wanted to maintain attachment and support; guilt when she has needs. Maintaining composure preserved safety and connection. ■ S: Mother’s move/flat-clearance and colleague’s illness: experiences charged with reminders of loss, aloneness, responsibility, and evaluation. These situations intensify her long-standing rule of composure, reviving the fear that experiencing or showing need, despair or anger could endanger her sense of safety and relationships. ■ E: Grief, anger, and guilt arise together; anger appraised as unsafe → turned inward; guilt polices attachment needs. ■ C: Holding in, perfectionistic checking, guarded closeness, narrowing of nourishing activities; attention lapses signal overload and indirect protest; tinnitus awareness rises on tense, avoided-feeling days. The resulting suppression heightens tension and inward focus, felt somatically as exhaustion, narrowing of interest, and nights filled with rumination. In this sense, Ms K’s distress is an understandable, meaning-laden response to conflicting emotional demands—attempts to stay in control and to protect attachment in a layered and complex situation that feels ambiguous and unsolvable.	Therapeutic principles (held, not rote; human, kind, professional): ■ No intervention without understanding (and formulation): co-create a narrative that considers how Ms. K’s ways of coping protect senses of attachment and control for her, whilst gently approaching the central dilemma at their core: the more she holds herself together, the lonelier and more burdened she feels. Therapy becomes a shared space to explore this paradox without urgency or judgment. ■ Keep a living formulation — the map of vulnerabilities, stressors, emotions, coping, and values is revisited as new experiences emerge; moments of progress and setback alike are information, not deviation. ■ Keep attachment & early meanings in view — explore how safety, love, danger, guilt, self-punishment, and internalised anger were learned; recognize that suppression once preserved closeness and stability; respect this function before inviting gentler alternatives when safety allows. ■ Work with ambivalence, not against it — approach conflicting impulses (wanting rest yet fearing uselessness, longing for support yet fearing burden) as meaningful signposts, not resistance. Help Ms. K notice how these cross-currents shape her choices and mood. ■ Safely hold nuanced emotion — create predictable and respectful opportunities to name and experience mixed emotional states (grief entwined with anger, tenderness beside fear) without shame or flooding. ■ Relational experiments with consent — ambivalence, guilt, or relief are welcomed as data for revising old expectations about care and dependence. ■ Meaningful re-engagement — return to activities that matter to Ms. K (connection, craft, music, movement with resonance), noticing how engagement might both awaken interest and stir mixed feelings of loss or hope.

DSM, Diagnostic and Statistical Manual of Mental Disorders; DSM-5-TR, Diagnostic and Statistical Manual of Mental Disorders, Fifth Edition—Text Revision; ICD, International Classification of Diseases (ICD-10; ICD-11); APA, American Psychiatric Association; WHO, World Health Organisation; HiTOP, Hierarchical Taxonomy of Psychopathology; VSEC, Vulnerability–Stress–Emotion–Coping; V, S, E, C, vulnerability, stress, emotion, coping; CBT, cognitive-behavioural therapy; nocte, at night; N-of-1, single-case time-series design.

† Spectra-level treatment guidance is under active study; owed to the recent development of the model, there is currently only little evidence that HiTOP-based psychological interventions improve outcomes over diagnosis-based treatment approaches.

‡ VSEC is not explanatory by fiat; it becomes explanatory only when meaning is assessed and formulated: (a) elicit personal meanings (including layered/conflicting/tacit beliefs); (b) specify functional links (what the behaviour/emotion helps the person with); and (c) test these links over time (e.g., brief N-of-1), revising accordingly.

Dominant medical diagnostic frameworks for tinnitus-correlated distress are nomothetic and descriptive. This contributes to a persistent conceptual difficulty in mental health care: the tendency to mistake psychiatric labels—used to describe symptom-patterns linked to emotional distress—for causal explanation. Terms such as “major depressive disorder” or “anxiety disorder” are often treated as if they denoted discrete quasi-medical pathologies which can explain a person’s emotional, cognitive, or behavioural states. In reality, diagnostic labels are classificatory terms that somewhat crudely summarise patterns of symptoms and behaviours, not the lived experience of distress itself. As such, they are better thought of as operational definitions rather than theoretically grounded constructs, and they capture only a narrow slice of psychological reality. This explanatory fallacy risks obscuring the importance of personal history, social context, and subjective meaning, reducing complex distress experiences to a presumed internal (and often pathologised) dysfunction.

Whilst such frameworks offer a widely shared clinical vocabulary, their scientific limitations—particularly regarding reliability, validity, and clinical utility—are well documented. Dimensional models such as the Hierarchical Taxonomy of Psychopathology (HiTOP) represent a meaningful advance, providing greater empirical precision and transdiagnostic coherence. Yet they remain population-level frameworks which do not aim to explain *why* a specific individual experiences (tinnitus-correlated) distress. Whilst descriptively useful, anchoring research and practice in systems that overlook individual meaning-making and psychological heterogeneity risks perpetuating conceptual blind spots that constrain both therapeutic innovation and scientific progress.

Idiographic approaches, such as the VSEC model, offer one (though not the only) corrective. By attending to individual developmental history, relational dynamics, and emotion regulation strategies, they facilitate a more contextualised understanding of distress. However, without integrating subjective meaning, such models risk becoming a flat “list-of-factors-conceptualisation” rather than a coherent, predictive explanatory narrative.

Only when subjective meaning is considered—i.e., when clinicians explore how individuals interpret and respond to their experiences and why—does the VSEC framework become explanatory. It then supports the construction of dynamic, psychologically grounded formulations that account for developmental processes, coping strategies, and context-mediated functions of symptoms.

Clinically, the VSEC model can guide collaborative assessment, foster therapeutic alliance, and enable tailored intervention targets—whether aimed at shifting broader maladaptive appraisals, strengthening coping repertoires in personally meaningful ways, or addressing unresolved relational difficulties. Importantly, the VSEC model is not a prescriptive protocol, but a flexible framework that supports clinical reasoning—guided by theory and grounded in the fluid, lived experience of the individual.

The implications are threefold:

1. Theoretically, advancing the science and practice of individualised psychosomatic care requires a shift in how chronic tinnitus itself is conceptualised. Rather than a standalone symptom or “disorder”, tinnitus is better understood as a contextually perceived and interpreted stimulus, emerging at the intersection of physiological bottom-up processes and dynamic top-down appraisals. Somatic mechanisms may contribute to initial symptom onset, but the persistence and distress associated with tinnitus are shaped by subjective appraisals and biographical meaning.
2. Clinically, it promotes individualised, collaborative care that respects how distress is embedded in personal context. As multidisciplinary services expand access—often by training non-psychologists (e.g., nurses or audiologists) in protocolised cognitive behavioural therapy (CBT) skills—there is both a chance of increased access or support options and well as a risk of equating psychological competence with manualised delivery. The core contribution of clinical psychology is meaning-focused formulation: eliciting layered personal meanings, specifying the functions of emotions and behaviours in context, and iteratively testing hypotheses over time.
3. Methodologically, it highlights the need for innovative mixed-method designs which are interested in and can capture meaning, complexity and change over time. The VSEC model offers a structured idiographic framework for exploring these interrelated dynamics, supporting a nuanced understanding of how tinnitus may become distressing for a particular individual, but not another. Although operationalising subjective meaning remains methodologically challenging, its investigation is crucial —informing interventions that are not only empirically grounded but also personally meaningful.

Clinicians and scientist-practitioners are invited to:

1. recognise that using psychiatric diagnoses as eligibility requirements for service access and funding may create a self-perpetuating evidence cycle that reflects the limits of the very categories they presuppose;
2. treat diagnosis and emerging dimensional nosologies as descriptive, not explanatory, entry points—rather than endpoints—of psychological understanding and treatment planning;
3. be familiar with, and draw on, nomothetic evidence whilst recognising that population means do not map well onto individuals (group-to-individual generalisability is limited) and use such evidence as a guide which needs to be tested and adjusted in light of each person’s lived context, meaning structure, and preferences;
4. make person-centred formulation (VSEC + meaning) routine in chronic tinnitus care;
5. de-reify psychiatric labels to avoid over-medicalising psychosocial distress;
6. align services and funding to reward formulation-informed targets (processes, meanings, functions), not merely diagnostic enrolment;
7. train clinicians to assess meaning, appraisal, and function (not merely checklist symptoms or mechanistically apply standardised “CBT” routines); and
8. advance mixed-methods research programmes that pair dimensional measures with qualitative meaning-mapping; deploy idiographic longitudinal designs (e.g. N-of-1 time-series) to model within-person coupling of tinnitus appraisals, affect, and coping; use process-based targets and network models to test causal hypotheses; and leverage large-scale language methods to index personal meaning from narratives;
9. most importantly, measure what matters: individual meanings linked to vulnerabilities and circumstances—despite operationalisation challenges— if genuine progress is to be made.

## Data Availability

The original contributions presented in the study are included in the article/supplementary material. Further inquiries can be directed to the corresponding author.

## Footnotes

1 Given their limited relevance to the psychosocial dimensions of tinnitus-correlated distress, we set aside medical–explanatory perspectives (both nomothetic and idiographic). While worthwhile to explore, these accounts currently contribute relatively little to understanding the psychosocial determinants and maintenance of tinnitus-correlated distress in clinical practice.
